# Identification of a Novel Proline-Rich Antimicrobial Peptide from *Brassica napus*

**DOI:** 10.1371/journal.pone.0137414

**Published:** 2015-09-18

**Authors:** Huihui Cao, Tao Ke, Renhu Liu, Jingyin Yu, Caihua Dong, Mingxing Cheng, Junyan Huang, Shengyi Liu

**Affiliations:** 1 Oil Crops Research Institute, Chinese Academy of Agricultural Sciences, Key Laboratory of Biology and Genetic Improvement of Oil Crops, Ministry of Agriculture, Wuhan, 430062, China; 2 Department of Life Science and Technology, Nanyang Normal University, Wolong Road, Nanyang 473061, China; 3 Hubei Collaborative Innovation Center for Green Transformation of Bio-Resources, Hubei University, Wuhan 430062, China; 4 Zhejiang Academy of Agricultural Sciences, 198 Shiqiao Road, Hangzhou, 310021, P. R. China; University of Groningen, NETHERLANDS

## Abstract

Proline-rich antimicrobial peptides (PR-AMPs) are a group of cationic host defense peptides that are characterized by a high content of proline residues. Up to now, they have been reported in some insects, vertebrate and invertebrate animals, but are not found in plants. In this study, we performed an *in silico* screening of antimicrobial peptides, which led to discovery of a *Brassica napus* gene encoding a novel PR-AMP. This gene encodes a 35-amino acid peptide with 13 proline residues, designated *BnPRP1*. BnPRP1 has 40.5% identity with a known proline-rich antimicrobial peptide SP-B from the pig. *BnPRP1* was artificially synthetized and cloned into the prokaryotic expression vector pET30a/His-EDDIE-GFP. Recombinant BnPRP1 was produced in *Escherichia coli* and has a predicted molecular mass of 3.8 kDa. Analysis of its activity demonstrated that BnPRP1 exhibited strong antimicrobial activity against Gram-positive bacterium, Gram-negative bacterium, yeast and also had strong antifungal activity against several pathogenic fungi, such as *Sclerotinia sclerotiorum*, *Mucor sp*., *Magnaporthe oryzae* and *Botrytis cinerea*. Circular dichroism (CD) revealed the main secondary structure of BnPRP1 was the random coil. *BnPRP1* gene expression detected by qRT-PCR is responsive to pathogen inoculation. At 48 hours after *S*. *sclerotiorum* inoculation, the expression of *BnPRP1* increased significantly in the susceptible lines while slight decrease occurred in resistant lines. These suggested that *BnPRP1* might play a role in the plant defense response against *S*. *sclerotiorum*. BnPRP1 isolated from *B*. *napus* was the first PR-AMP member that was characterized in plants, and its homology sequences were found in some other Brassicaceae plants by the genome sequences analysis. Compared with the known PR-AMPs, BnPRP1 has the different primary sequences and antimicrobial activity. Above all, this study gives a chance to cast a new light on further understanding about the AMPs’ mechanism and application.

## Introduction

Antimicrobial peptides (AMPs) are a group of diverse endogenous antibiotics and innate immune components that protect hosts against microbial infection and are produced ubiquitously in the natural environment. Hundreds of antimicrobial peptides have been isolated from plants, mammals, insects, bacteria, fungi and so on [1]. The peptide structures identified to date can be classified into five major classes: α-helical, cysteine-rich (defensin-like), β-sheet, peptides with an unusual composition of regular amino acids, and peptides that contain uncommon modified amino acids [2]. In addition, AMPs usually have common features such as a small molecular weight (<10 kDa), a cationic surface, a positive charge, and amphiphilicity [3], these properties make AMPs easily and rapidly permeabilize microbial membranes to ‘kill’ the microbe. Most AMPs might nonspecifically interact with microbial membranes, leading to subsequent membrane damage and microbial lysis through ‘lytic’ mode mechanisms [4,5].

Unlike other types of AMPs, Proline-rich antimicrobial peptides (PR-AMPs) act via distinctive ‘non-lytic’ mechanisms [6–8]. PR-AMPs penetrate into microbial membranes without disrupting membranes integrity, and then bind to and interact with the specific intracellular target(s). These features make them attractive for both basic and applied research efforts, and provide novel insights into the mechanism of action of anti-infection chemical agents in both bacterial and eukaryotic cells.

As a large group of linear peptides, PR-AMPs were reported first in honeybees [9], and were identified subsequently in species including mammals [7], amphibians [10], crustaceans [11], and molluscs [12]. PR-AMPs generally refer to peptides with more than 30% proline residues in their primary structure.

Plant AMPs can be assigned to different groups according to their specific structural characteristics, such as thionins, defensins, lipid transfer proteins, cyclotides, and snakins [13–16]. However, PR-AMPs have not been reported in plants to our knowledge. In an effort to search for new AMPs using large available genome and expression databases, we found a series of candidate AMPs. Of the AMPs identified, a unusual plant peptide BnPRP1 containing 13 prolines in its 35 amino acid sequence, which was similar to SP-B (a PR-AMP from pig) [17], was studied functionally.

## Materials and Methods

### Plant material and microbial strains

Six *B*. *napus* lines were used: Zhongshuang 9, Zhongshuang 11, Zhongyou 821 and M083, which are resistant to *S*. *sclerotiorum*, and 888–5 and 84039, which are highly susceptible to *S*. *sclerotiorum*. All the *B*. *napus* lines used in this study were grown in a greenhouse under a 16/8 h (day/night) cycle at 22 ± 2°C in the Oil Crop Research Institute of the Chinese Academy of Agricultural Sciences.

Two bacterial strains and five fungal strains were used for activity analysis: the Gram-negative bacterium *Escherichia coli* ATCC25922, the Gram-positive bacterium *Micrococcus luteus* ACCC11001, and the fungi *Pichia pastoris* GS115, *S*. *sclerotiorum*, *Mucor sp*., *M*. *oryzae* and *B*. *cinerea*. All strains were stored frozen in 20% glycerol at −80°C. *E*. *coli* XL10-GOLD and *E*. *coli* BL21 (DE3) were used for plasmid construction and recombinant protein expression, respectively. The plasmid pET30a/His-EDDIE-GFP [18] was used to construct the AMPs expression vector. These strains were developed or introduced by the Oil Crop Research Institute of the Chinese Academy of Agricultural Sciences (Wuhan City, China).

### Identifying candidate gene *BnPRP1*

*B*. *napus* EST sequences from our own database and the GenBank EST database downloaded were BLASTed against known AMPs sequences that had been identified and characterized previously in AMP databases, such as ANTIMIC [19], APD2 [20], and PhytAMP [21]. Approximately 606 AMP candidate genes were obtained from these *B*. *napus* EST sequences (data not shown). One of these candidate AMP genes, named *BnPRP1*, which encoded a peptide sequence with high proline content, was identified from *B*. *napus* etiolated seedlings EST sequence (GenBank Accession No. EV168192).

### Phylogenetic analysis

The amino acid sequences of all PR-AMPs genes, which were obtained from the protein database at the National Center for Biotechnology Information, the antimicrobial peptides database (http://aps.unmc.edu/AP/main.php), database for *Brassica rapa* (http://brassicadb.org/brad),database for *Brassica oleracea* (http://www.ocri-genomics.org/bolbase) and database for *Brassica napus* (http://www.genoscope.cns.fr/brassicanapus/), were aligned using ClustalW (version 1.8) program [22] with default parameters. The phylogenetic tree was constructed using MEGA software version 5.0 [23] via the neighbor-joining method with 1000 bootstrap replicates.

### Construction of an expression vector expressing Recombinant protein fused to EDDIE

According to a method described previously [18], *BnPRP1* nucleotide sequence was optimized according to *E*. *coli* codon usage, then assembled using primers (Table 1) in a one-step PCR reaction: 25 cycles of 94°C for 30 s, 58°C for 30 s, and 72°C for 10 min using Pyrobest^TM^ DNA polymerase (Takara Bio Inc., Japan). The pET30a/His-EDDIE-GFP vector was amplified and linearized using the primers backboneF and backboneR. The PCR reaction was performed with 25 cycles of 94°C for 30 s, 60°C for 30 s, and 72°C for 7 min using Pyrobest^TM^ DNA polymerase (Takara Bio Inc., Japan). The synthetic *BnPRP1* and the linearized vector were co-transformed into *E*. *coli* XL10-GOLD. When the target gene cloned into the vector, the GFP gene of pET30a/His-EDDIE-GFP vector was destroyed and white colonies were picked under ultraviolet light. The resulting BnPRP1 expression vector was named pET30a/His-EDDIE-BnPRP1.

**Table 1:** Sequences of the primer used for overlap-PCR and qRT-PCR.

Experiment	Primer name	Primer sequence (5'->3')
Overlap-PCR	BnPRP1 _1	**CGCTGTGGGTGACCAGC**TGCGCCGCCGACCCAAAATCCGAGC
	BnPRP1 _2	AGGCTGGCCGTAAGGGTTCTGAGTTGGAGGCGCCATGCTCGGATTTTGGGTCGG
	BnPRP1 _3	CCCTTACGGCCAGCCTATGGCACCCCCTACCCAGAATCCGTATGGGCAGCCGAT
	BnPRP1 _4	**GGGCTTTGTTAGCAGCCGGATCTCA**CGGTGGAGCCATCGGCTGCCCATACGG
Vector amplify	backboneF	TGAGATCCGGCTGCTAACAAAGCCC
	backboneR	GCAGCTGGTCACCCACAGCG
RT-PCR	5'-BnPRP1	CTACCCAAAATCCGAGCATGG
	3'-BnPRP1	CATACGGATTCTGGGTAGGG
	5'-actin	CTGGAATTGCTGACCGTATGAG
	3'-actin:	ATCTGTTGGAAAGTGCTGAGGG

The plasmid pET30a/His-EDDIE-BnPRP1 was transformed into *E*. *coli* BL21 (DE3) cells. A single colony was picked, inoculated into 50 mL LB (1% tryptone, 0.5% yeast extract, 1% NaCl) medium with 50 μg/mL kanamycin, and grown in a shaking incubator in 37°C overnight. Next morning, 50 mL fully grown culture was added to 1 L LB medium with 50 μg/mL kanamycin and grown in shaking incubator at 25°C. When the OD_600_ reached to 0.5, IPTG was added to a final concentration of 1 mM. The culture cells were harvested and then washed and resuspended in PBS buffer (sodium phosphate buffer: 137 mM NaCl, 2.7 mM KCl, 4.3 mM Na_2_HPO_4_, 1.4 mM KH_2_PO_4_, pH 7.2–7.4).

### Purification of fusion protein, refolding, and peptide purification

The harvested bacterial cells described above were lysed by ultrasonication, following the manufacturer’s instructions (Sonics uibracell, Sonics & Materials, Inc., USA) as output watts of 200 W for 6min, amplitude of 60% and pulse durations of 6 s ON and 6 s OFF, and the insoluble inclusion bodies were harvested by centrifugation at 14,000 g for 30 min in 4°C. The pellet was then washed three times with washing buffer (10 mM Tris-HCl pH 7.6, 200 mM NaCl, 1% Triton X-100, and 2 mM 2-mercaptoethanol), and solubilized in denaturing buffer (8 M urea, 20 mM Tris-HCl pH 7.6, and 5 mM 2-mercaptoethanol) for 1 h.

The purified inclusion bodies His-EDDIE-BnPRP1 were refolded by rapid dilution 1:50 in optimized refolding buffer (500 mM NaCl, 20 mM Tris, 2 mM EDTA, 5% glycerol, 10 mM DTT and 0.01% Tween-20 pH 7.5) and incubated at 22–25°C without stirring. After BnPRP1 was released from the fusion protein by EDDIE self-cleaving at a specific site, the renatured protein solution was then clarified by centrifugation at 15,000g for 30 min at 4°C. Any insoluble particles were removed by filtering through a 0.45-μm membrane, and the supernatants were applied to a Ni-NTA His-bind column and BnPRP1 were left in the supernatant [18]. SDS-PAGE on 12% gels was then used to separate the fusion proteins. Tricine denaturing polyacrylamide gel electrophoresis (Tricine-SDS-PAGE) was performed according to Schagger and Von Jagow [24] with a 12% separating gel and a 4% stacking gel. The gel was stained with 0.25% (w/v) Coomassie brilliant blue R-250 in 45% methanol and 10% acetic acid.

### Chemical synthesis and purification of *BnPRP1* peptide

BnPRP1 peptide was synthesized according to its putative amino acid sequence without any modifications using Fmoc (N-[9-fluorenyl] methoxycarbonyl) chemistry by the Basic Science Institute of Wuhan Bioyeargene Biosciences Co. Ltd (Wuhan, China). The synthetic peptide was purified (>95% homogeneity) using reverse-phase HPLC on a C18 column (4.6 × 250 mm, Delta Pak, Waters) with a linear gradient of 5–45% acetonitrile in 0.05% trifluoroacetic acid for 25 min. All peptides were characterized using matrix assisted laser desorption ionization mass spectroscopy (MALDI-TOF; AB SCIEX), and the peptide content of the lyophilized samples was determined according to quantitative amino acid analysis with a Pico-tag analysis system on a Beckman 121 MB amino acid analyzer (Beckman Coulter).

### Activity analysis

The antimicrobial activity of BnPRP1 was assessed using *M*. *luteus* (Gram-positive, G^+^), *E*. *coli* (Gram-negative, G^−^), and *P*. *pastoris* GS115 (yeast) as substrates in a radial diffusion assay [25]. 1.5% Broth agar (LB medium for bacteria, and YEPD medium containing 2% tryptone, 1% yeast extract, 2% glucose, 1.5% Agar for *P*. *pastoris*) containing the tested strain (OD_600_ = 0.1) was poured onto 90 mm plates. The purified recombinant and chemically synthesized BnPRP1 peptide samples were diluted to the same concentrations (3 mg/mL), placed into individual wells in the agar plates, and incubated at 37°C or 28°C for 16 h, respectively. Refolding buffer was used as the negative control. The diameters of the lysed circular zones were then measured. The above assays were performed in triplicate.

The antifungal activity of the purified products was assayed using an ultra-sensitive radial diffusion method on thin potato plates (200 g potato, 20 g glucose, 15–20 g agar powder, and 1 L double-distilled water) seeded with filamentous fungi [26]. Briefly, 90 mm plates were poured on an underlay potato medium, and *S*. *sclerotiorum*, *M*. *oryzae*, *Mucor sp*. and *B*. *cinerea* were seeded on the center of the plates. The plates were then incubated at 22°C for *S*. *sclerotiorum* and *M*. *oryzae*, 25°C for *Mucor sp*. and 28°C *for B*. *cinerea* until the filamentous fungi grew to 2 cm in diameter. Two hundred microliters of the test sample (3 mg/mL) was placed beside the filamentous fungi, and the plates were incubated at 22°C, 25°C, or 28°C for 72 h, respectively; the size of the clear area around the filamentous fungi was then measured. The inhibition of *S*. *sclerotiorum* growth by BnPRP1 was also examined under an inverted system microscope (IX71 Olympus, Japan) after incubation at 22°C for 48 h.

### Antimicrobial MIC assays

The lowest concentration of peptide that inhibited the growth of the organisms completely was defined as the minimal inhibitory concentration (MIC). The MIC of BnPRP1 against Gram-positive and Gram-negative bacteria was determined using broth microdilution assays. Briefly, single bacterial colonies were inoculated into culture medium (1% yeast extract, 2% tryptone, and 2% NaCl) and cultured overnight at 37°C. One milliliter of this culture was transferred to 50 mL of fresh medium and incubated for an additional 3–6 h at 37°C to obtain mid-logarithmic phase cells. They were then harvested by centrifugation at 12,000 ×g for 15 min, then washed with 10 mM PBS, pH 7.4, and resuspended in 10 mL of fresh PBS. The number of colony-forming units (CFUs) per milliliter was determined by spreading serial dilutions of the cell suspension onto three separate trypticase agar plates. A two-fold dilution series of peptides in 10 mM PBS was prepared, and serial dilutions (50 μL) were added to 50 μL of 5 × 10^4^ CFU in static 96-well microtiter plates. After incubation for 3 h at 37°C, fresh medium was added to the mixture, and cells were incubated at 37°C for 16 h. Growth inhibition was determined by measuring the absorbance at 620 nm using a Wallac Victor-1420 microplate reader (Perkin-Elmer Life Sciences, Boston, MA, USA). The MICs were defined as the mean values obtained from triplicate samples on three independent measurements [27].

### Circular dichroism

Circular dichroism (CD) spectra were recorded by Chirascan (Applied Photophysics, Ltd) to determine the secondary structure of BnPRP1. The spectra were measured between 200 nm and 250 nm in different solvents as follows: water, 50 mM PBS, ethanol, methanol, and a concentration gradient of 25%, 50%, and 75% (vol/vol) of trifluoroethanol in 50 mM PBS. Consecutive scans were performed in a 1mm cell at 25°C. All measurements were conducted using peptide concentrations of 0.10 mg/mL in 10 mM potassium phosphate buffer (pH 7.4). The helicity of the peptide was determined from the mean residue helicity at 220 nm. The percentage of α-helix was calculated using the formula [θ]_222_ = −30,300f_H_− 2340 [28]. All data presented are the means of three independent measurements.

### Plant inoculation and gene expression analysis

The agar plugs (5mm in diameter) were excised from the edge of growing mycelia of *S*. *sclerotiorum*, and then upended onto the adaxial surface of plant leaves at the four- true-leaf stage. The inoculated plants were incubated under the dark condition at 22°C until sampling at 48h after inoculation.

Total RNA was extracted from the respective frozen tissues (stems, leaves and leaves inoculated with *S*. *sclerotiorum*) by using an RNeasy Plant Minikit (Qiagen, USA). DNA-free total RNA (1 μg) was reverse transcribed using reverse transcriptase (Invitrogen) following the manufacturer’s instructions. qRT-PCR was performed using SYBR Green Real-time PCR Master Mix (Bio-Rad, USA) with 0.8 μL of each primer (10 μM) and 2 μL 1:20 diluted cDNA template in 20 μL reaction mixture (CFX96, Bio-Rad). The gene-specific primers 5′-BnPRP1 / 3′-BnPRP1 and 5′-actin / 3′-actin were designed to amplify *B*. *napus BnPRP1* and *ACTIN* gene, respectively (Table 1). The PCR cycling conditions were as follows: 94°C of denaturation for 10 min, followed by 40 cycles of 94°C of denaturation for 30 s, 58°C of annealing for 30 s, and 72°C of extension for 30 s. At the end of each PCR reaction melting curve analysis was used to confirm that only one product was amplified and detected. The cycle threshold (Ct) values were used to calculate the fold changes in expression. The experiments were performed using three biological replicates. The relative expression of *BnPRP1* was calculated using the relative 2^-ΔCt^ method.

## Results and Discussion

### *In silico* screening and discovery of a novel PR-AMP

To identify potential antimicrobial peptides, first batch of sequences (i.e. *B*. *napus* ESTs generated in our lab and downloaded from GenBank) were aligned with known AMP sequences. A total of 606 genes were identified as the potential AMPs candidates. Among these was one gene that contained a 105 bp open reading frame (ORF) which encoded a peptide containing 13 prolines (37% of the total residues) within its 35 amino acid sequence. Proline-rich antimicrobial peptides (PR-AMPs) are characterized by a high content of proline, typically ranging from 25 to 50%, and so this peptide was named BnPRP1. BnPRP1 only shared similarity with four known PR-AMPs, in which SP-B (with 40.5% identity) isolated from the porcine salivary gland granules [17], BacFL31 (with 36.11% identity) identified from *Enterococcus faecium* [29], Abaecins isolated from bumblebee (with 34.09% identity) and honeybee (with 33.33% identity) [30,31]. BnPRP1 was the first reported proline-rich antimicrobial peptide in plants.

**Fig 1:**
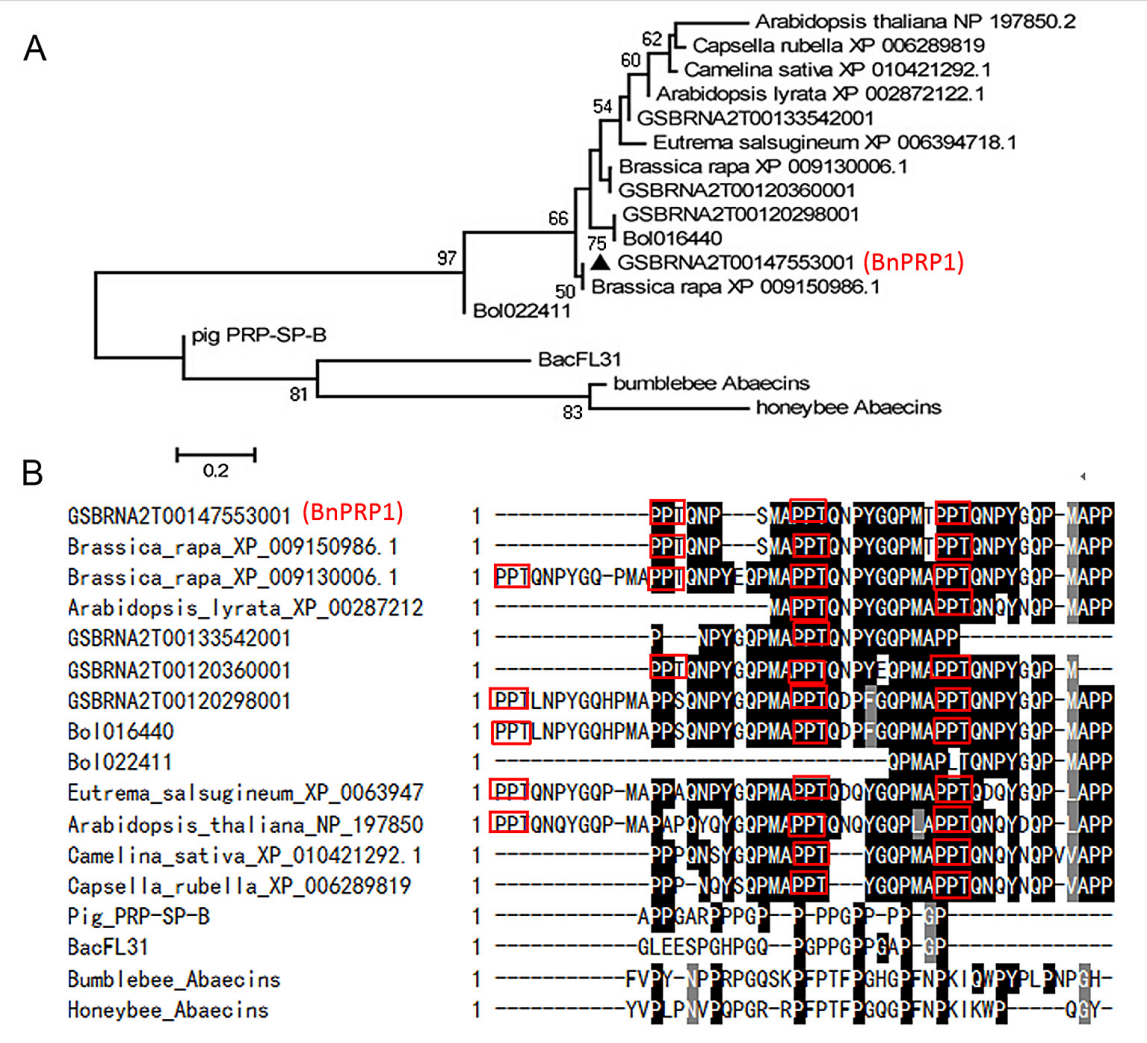
Multiple sequence alignment and phylogenetic analysis of *BnPRP1*. (A) Unrooted Phylogenetic tree of BnPRP1 and its homologous proteins. Dendrogram obtained using neighbor-joining analysis based on the proportion (p-distance) of aligned amino acid sites of the full-length peptide sequences. BnPRP1 is marked with a solid triangle. Numbers at the base of each clade correspond to the bootstrap means of 1000 replications. Organism’s common or taxonomic names are shown in parenthesis. The Genbank accession numbers/Database accession numbers for the proteins/peptides are as follows: *Arabidopsis thaliana* (NP_197850.2); *Capsella rubella* (XP_006289819); *Camelina sativa* (XP_010421292.1); *Arabidopsis lyrata* (XP_00287212); *Eutrema salsugineum* (XP_0063947); *Brassica napus* (*Brassica napus* Database accession No. GSBRNA2T00120298001); *Brassica oleracea* (bolbase accession No: Bol016440); *Brassica rapa* (brad accession No: XP_009130006.1); *Brassica napus* (*Brassica napus* Database accession No:GSBRNA2T00147553001); *Brassica rapa* (brad accession No: XP_009150986.1); *Brassica napus* (GSBRNA2T00133542001); *Brassica oleracea* (bolbase accession No: Bol022411); *Brassica napus* (*Brassica napus* Database accession No: GSBRNA2T00120360001); SP-B (APD accession No: AP00889); BacFL31(APD accession No: AP02346); bumblebee_Abaecins (APD accession No: AP01215); honeybee_Abaecins (APD accession No: AP00002). (B) Multiple alignments of BnPRP1 and its homologs. Multiple alignments of BnPRP1 and its homologs identical amino acid residues were black-shaded, PPT motif of BnPRP1 is shown in box.

In addition to these four known proline-rich antimicrobial peptides, the homology sequences of BnPRP1were only found in Brassicaceae plants by researching in GenBank and other sequenced genomes databases (Fig 1), such as *Arabidopsis thaliana*, *B*. *rapa*, *B*. *oleracea*, *B*. *napus*, *Capsella rubella*, *Camelina sativa*, *Arabidopsis lyrata*, *Eutrema salsugineum* and so on. Among these homologous sequences (shared 66%-97% identity with BnPRP1), there are four homologous sequences in *B*. *napus* (AACC, 2n = 38), two homologous sequences in *B*. *rapa* (AA, 2n = 20) and in *B*. *oleracea* (CC, 2n = 18), one homologous sequences in *A*. *thaliana*. However, no functional verification experiments have been performed for these putative PR-AMPs from the plants.

As Fig 1A showed, phylogenetic analyses based on protein sequence of BnPRP1indicate that BnPRP1 is located in one clade together with its homology sequences that come from Brassicaceae plants and the other clade contain the four known PR-AMPs (SP-B, BacFL31, Abaecins from bumblebee and honeybee). A ClustalW comparison revealed that all PR-AMPs homologous sequences from the plants shared highly similarity and contain conserved PPT repeated motif (Fig 1B).

### The synthesis of *BnPRP1*and construction of an expression vector

**Fig 2:**
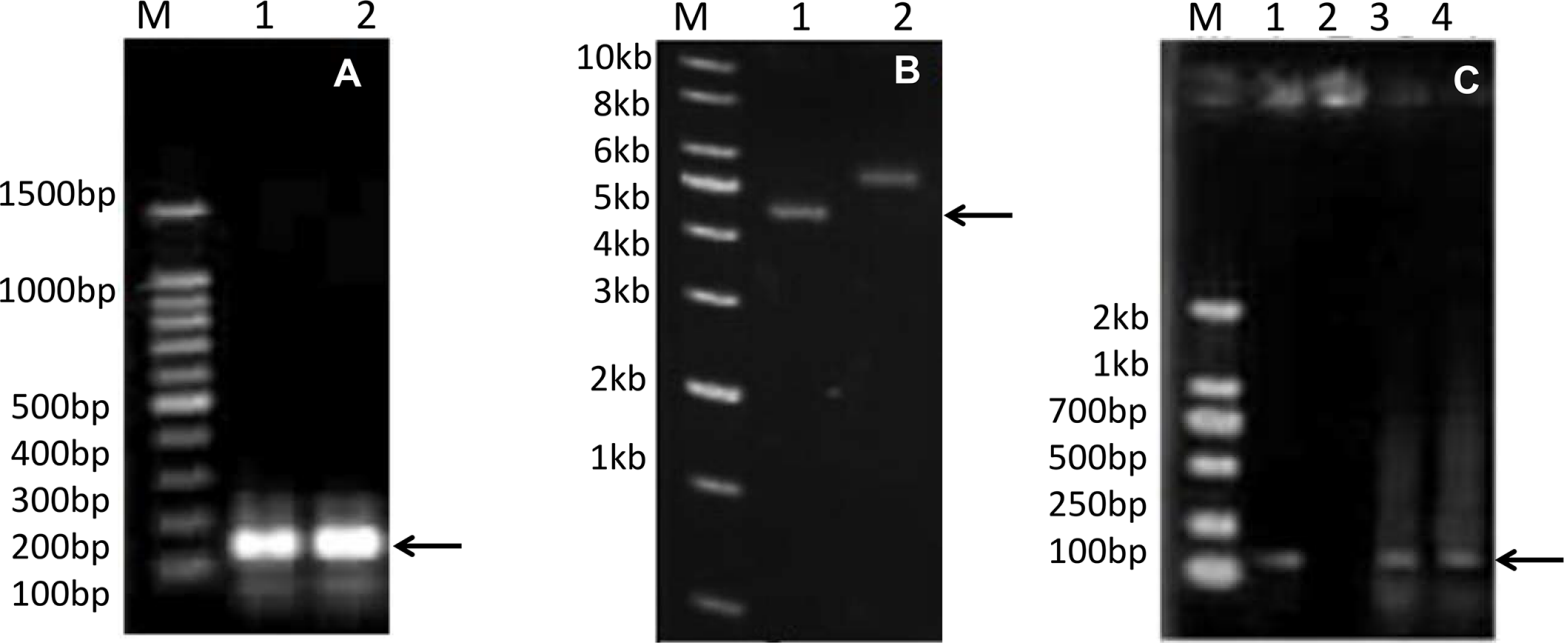
Agarose gel electrophoresis detection of the overlap PCR product from the *BnPRP1* gene and the recombinant plasmids pET30a/His-EDDIE-GFP and pET30a-EDDIE-BnPRP1. (A) Lane M, 100 bp DNA marker; Lanes 1–2, Overlap PCR products from the *BnPRP1* gene. (B) Lane M, 1 kb DNA marker; Lane 1, recombinant vector pET30a/His-EDDIE-BnPRP1; Lane 2, vector pET30a/His-EDDIE-GFP. **C:** Lane M, DL2000 DNA marker; Lane 1, positive control; Lane 2, negative control; Lanes 3 and 4, PCR detection of the *BnPRP1* gene.

The *BnPRP1* gene was assembled using four primers in a single PCR reaction (Fig 2A); the pET30a/His-EDDIE-GFP vector was replicated concurrently. The two PCR products were then transformed into *E*. *coli* together and assembled *in vivo* by homologous recombination. After screening white colonies under ultraviolet light and verification by PCR and sequencing (Fig 2C), the recombinant pET30a/His-EDDIE-BnPRP1 plasmid (Fig 2B) was constructed.

### Expression and purification of fusion proteins and refolding

**Fig 3:**
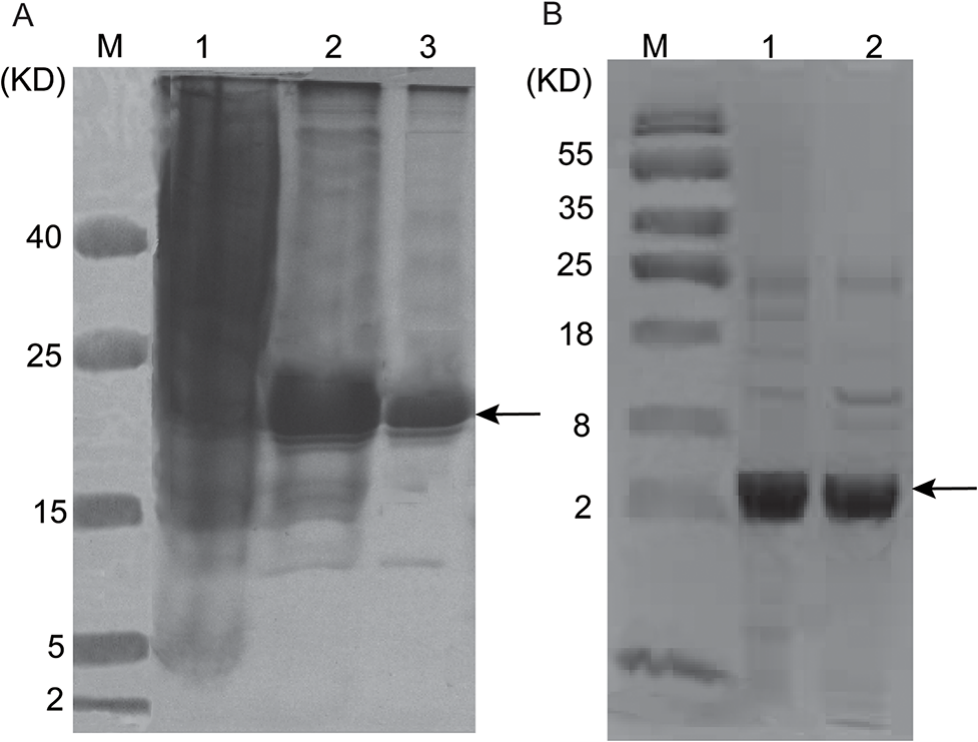
SDS-PAGE analysis of recombinant His-EDDIE-BnPRP1 expressed in *E*. *coli* BL 21 (DE3) and Tricine-SDS-PAGE analysis of BnPRP1 renaturation. (A) SDS-PAGE analysis of recombinant His-EDDIE-BnPRP1. Lane M, protein molecular weight maker; Lanes 2, precipitation of His-EDDIE-BnPRP1; Lane 1, *E*. *coli* BL 21 (DE3) cells transformed with pET30a plasmid as a control. Lane 3, purified His-EDDIE-BnPRP1. (B) Tricine-SDS-PAGE analysis of BnPRP1 renaturation. Lane M, protein molecular weight marker; Lanes 1, 2, refolded BnPRP1 after 8 h.

The pET30a/His-EDDIE-BnPRP1 plasmid was transformed into *E*. *coli* BL21 (DE3) cells and the recombinant bacteria were induced to express the His-EDDIE-BnPRP1 fusion protein using IPTG. SDS-PAGE revealed that fusion proteins sized ~23 kDa represented the majority of the insoluble components in the cell lysates (Fig 3A). Purified His-EDDIE-AMP inclusion bodies were then diluted in optimized refolding buffer and incubated to enable self-cleavage. After refolding, the EDDIE fusion partner was removed using Ni-NTA His column chromatography to leave the purified BnPRP1 in the solution. The results of Tricine-SDS-PAGE showed that BnPRP1 had a predicted molecular mass of 3.8 kDa and was self-cleaved efficiently from EDDIE after refolding (Fig 3B).

### Activity analysis

**Fig 4:**
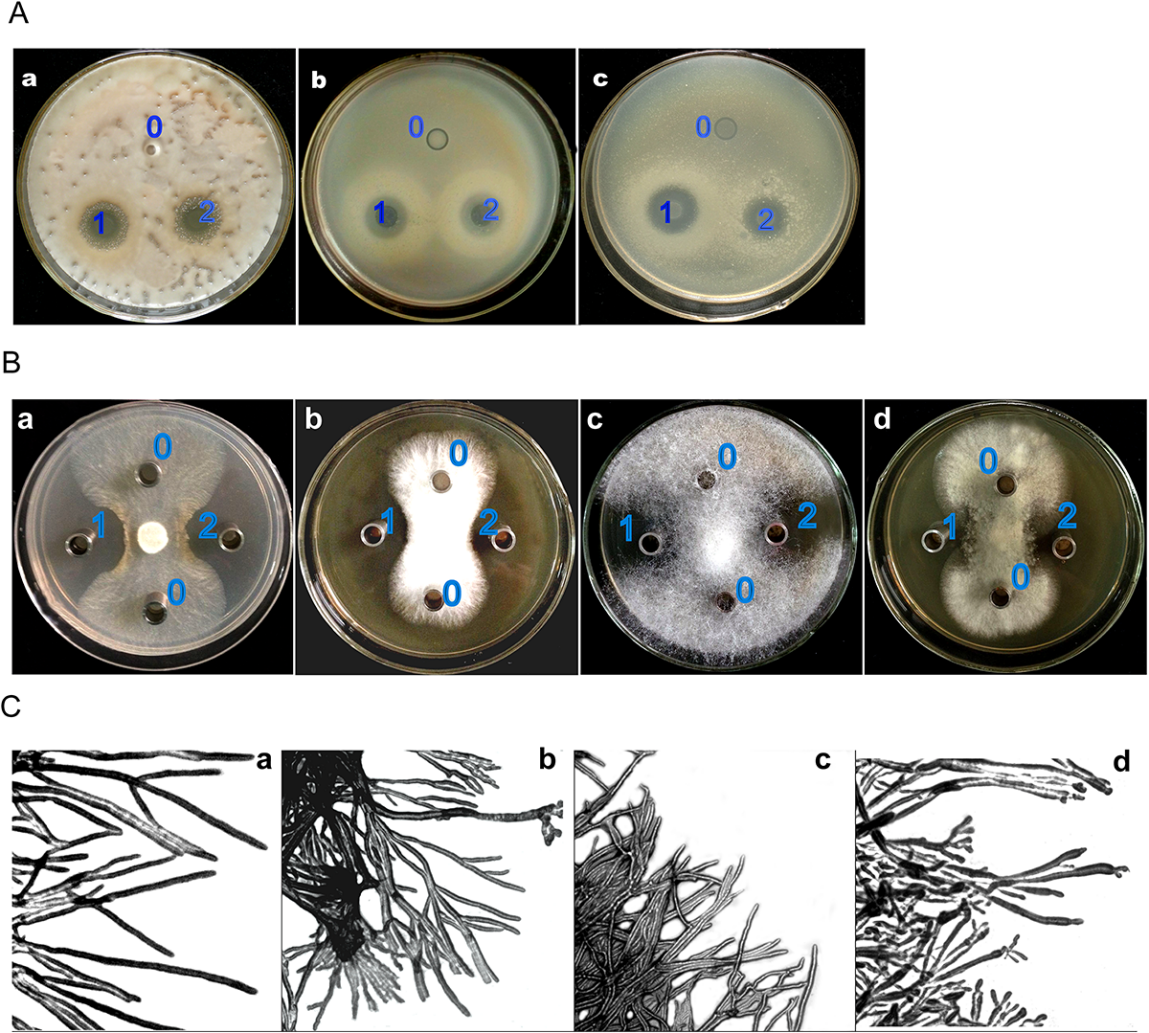
*BnPRP1* peptide activity inhibiting bacteria and fungi. (A) Assay of the BnPRP1 peptide activities against *E*. *coli*, *M*. *luteus*, and *Pichia pastoris*. a: *P*. *pastoris*; b, *M*. *luteus*; c, *E*. *coli*; 0, refolding buffer; 1, purified synthetic BnPRP1; 2, purified natural BnPRP1. (B) Assay of the BnPRP1 peptide activities against fungi. a, *S*. *sclerotiorum*; b, *Mucor sp*.; c, *M*. *oryzae*; d, *B*. *cinerea*, (C) Light microscopic examination of the activity of BnPRP1 against *S*. *sclerotiorum*. a, normal hyphae; b, knotted hyphae; c, Hyphae adhesion; d, The top of the hyphae knot.

The antimicrobial activity of the chemically synthetic and purified recombinant BnPRP1 against seven selected organisms, including Gram-positive, Gram-negative bacterium, yeast, and fungi, was determined using Oxford plate assays. As shown in Fig 4A, large halos were present around the synthetic and recombinant BnPRP1, indicating that BnPRP1 has specific inhibitory activities against *E*. *coli*, *M*. *luteus*, and *P*. *pastoris*. In contrast, no inhibitory zones were seen around the negative control spots. The results of activity detection demonstrated that synthetic and recombinant BnPRP1 exhibited the same strong inhibitory activity against Gram-positive bacterium, Gram-negative bacterium and yeast.

Four agronomical important fungal pathogens strains were selected to assay the anti-fungal activities of BnPRP1: *S*. *sclerotiorum*, *Mucor sp*., *M*. *oryzae* and *B*. *cinerea*. The results revealed that the same concentration of both synthetic and recombinant BnPRP1 exhibited strong activity to inhibit the growth of all four fungi. In addition, the hyphal growth of the fungal strains was not inhibited in the absence of peptide (control), and hyphae could bypass the inhibition zone and continue to spread to the edge of the plate (Fig 4B). The morphology of the restricted fungal hyphae was also altered by treatment with BnPRP1. Compared with the control cultures, shorter and somewhat more branched hyphae were observed when BnPRP1 was included in the growth medium (Fig 4C). The morphological changes also included abnormal branching, hyphal swelling, and knotting.

### The minimal inhibitory concentration (MIC) of *BnPRP1*

The MIC of BnPRP1 was 8.4 ± 1.4 μM (31.25 ± 5 μg/mL) against *E*. *coli*, and 16.9 ± 2.7 μM (62.5 ± 10 μg/mL) against *M*. *luteus* (Table 2). The antimicrobial activities of the well-characterized PR-AMPs from insect and mammalian were assessed previously. Apidaecins, which are the earliest insect PR-AMPs derived from bees, were active against both Gram-positive and Gram-negative bacteria and had an MIC of 25–50μg/mL against *E*. *coli* NCTC9001 [9, 30, 32, 33]. Another well-known member of the PR39 isolated from pig intestine exhibited antibacterial activity against *E*. *coli* ATCC25922 and Gram-positive bacteria, both with an MIC of 20 μg/mL [34,35]. SP-B which has the best similarity with BnPRP1 possessed strong antifungal activity but only negligible antibacterial activity [17]. BnPRP1 not only exhibited strong activity against Gram-negative and Gram-positive bacteria, but also had strong antifungal activity.

**Table 2:** Minimal concentrations of *BnPRP1* required for complete growth inhibition.

Microorganism	Minimal inhibitory concentration (μM)^a^
*M*. *luteus* ACCC11001	16.9± 2.7
*E*. *coli* ATCC25922	8.4 ±1.4

^a^Results are the mean values obtained from three independent measurements.

### Circular dichroism

**Fig 5:**
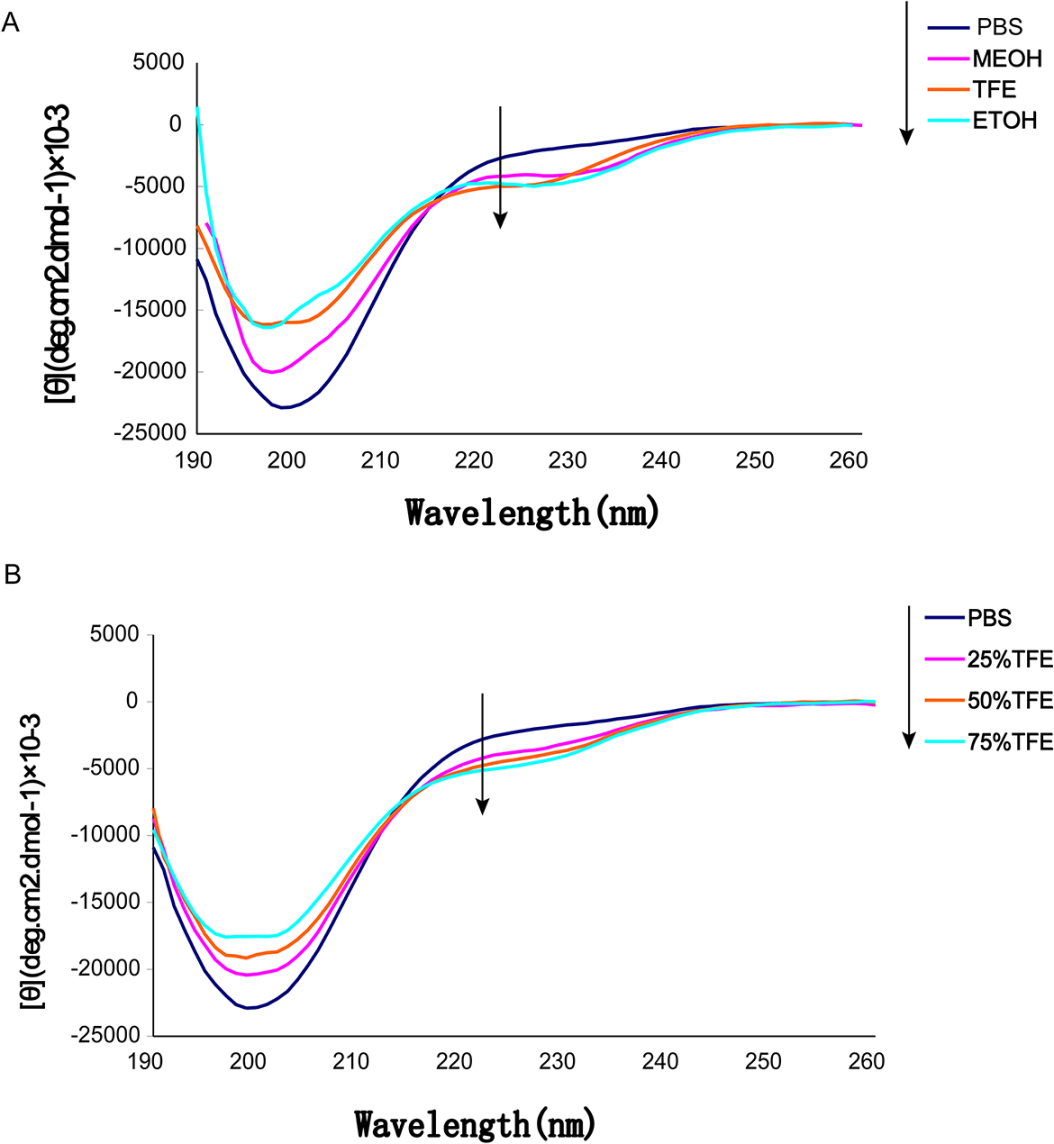
CD spectra of *BnPRP1* in different solvents (A) and in the presence of 0–75% (v/v) TFE (B).

Circular Dichroism (CD) was performed to obtain information regarding the secondary structure of BnPRP1. The chemically synthetic BnPRP1 was dissolved in different solvents: double-distilled water, PBS, methanol (MeOH), ethanol (EtOH), and different concentrations of trifluoroethanol (TFE). Fig 5A shows the CD spectra of BnPRP1 in different solvents in 190–250nm ultraviolet light. BnPRP1 mainly formed a random coil structure, and only a small amount of α-helix content was present, as shown in Table 3 and calculated according to method described by Chen *et al*. [28]. However, the CD or nuclear magnetic resonance (NMR) study of many mammalian and insect PR-AMPs showed that they tend to form the poly-L-proline II helix (PP-II helix) [6–8]. Interactions between solvent molecules and the hydrophobic groups of peptides, alcohols, and particularly TFE can induce the formation of helical structure effectively [36]. To obtain detailed information regarding the effect of organic solvent on the secondary structure of BnPRP1, three different concentrations of TFE in PBS solution were used to study the effect of organic solvents on its secondary structure. As shown in Fig 5B, the CD spectrum of BnPRP1 yielded a strong negative band at 200 nm in PBS, which was indicative of a random-coil conformation. Interestingly, in TFE/PBS mixtures the CD spectra exhibited double minima at 200 nm and 222 nm, indicating that TFE induced the formation of a helical structure. In addition, an increasing concentration of TFE enhanced the strength of the peak, which indicated an increased helical structural content. When BnPRP1 was dissolved in aqueous buffer and PBS, no α-helix was observed. However, in the presence of 30%, 50%, and 70% TFE BnPRP1 exhibited 0–10% α-helix content.

**Table 3:** Structural data of *BnPRP1* obtained from CD.

Solution^a^	50 mM PBS	25% TFE^+^in50 mM PBS	50% TFE^+^in50 mM PBS	75% TFE^+^in50 mM PBS	MeOH	TFE	EtOH
% α-helix	1.5	6.3	8	9.2	6	8.8	8

^a^PBS, pH 7.4

TFE^+^, trifluoroethanol; MeOH, methanol; EtOH, ethanol

### Response of *BnPRP1* Expression to *S*. *sclerotiorum* inoculation

**Fig 6:**
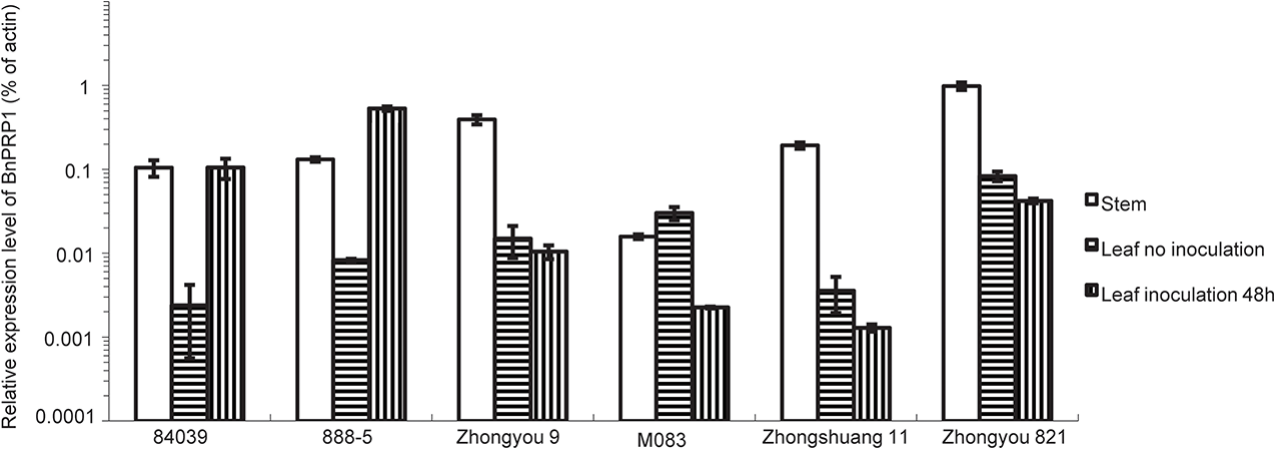
Response of *BnPRP1* Expression to *S*. *sclerotiorum* inoculation. Quantitative real-time PCR was used to measure *BnPRP1* gene expression in the leaves and stems of six *Brassica napus* cultivars: four disease-resistant (Zhongshuang 9, M083, Zhongshuang 11, and Zhongyou 821) and two susceptible cultivars (84039 and 888–5). All values are the means obtained from three biological replicates and their respective standard deviations.

To test whether BnPRP1 expression responses to pathogen inoculation, qRT-PCR was used with RNA samples prepared from stems, leaves and *S*. *sclerotiorum*- inoculated leaves of six *B*. *napus* lines. Compared with non-inoculated plants, the expression of BnPRP1 was significantly up-regulated 48 h after *S*. *sclerotiorum* inoculation in the susceptible lines 84039 and 888–5, while slightly decreased in the resistant varieties Zhongyou 9, M083, Zhongshuang11 and Zhongyou821 (Fig 6). These suggested that BnPRP1 may be involved in the plant defense response against *S*. *sclerotiorum*. But there is a need to measure BnPRP1expression at early time (e.g. 12h or 24h) to check whether the reversely induced effects occur when considering that many genes in resistant lines response more dramatically. Furthermore convincing results should be those from test of transgenic plants in which genetic difference is just due to this gene.

## Conclusions

In the current study, a novel gene *BnPRP1* was identified from *B*. *napus* using bioinformatics methods. The activity analysis results showed that BnPRP1 exhibited a strong and broad spectrum antimicrobial activity against bacteria and fungi. CD experiments showed that BnPRP1 predominantly exhibits a random-coiled structure. These results showed that BnPRP1 might be a novel member of the proline-rich antimicrobial peptide family with characteristics that distinguish it from the known PR-AMPs. Up to now, it is the first PR-AMP which was predicted and experimentally confirmed in plants. Above all, these features make BnPRP1 attractive in the further study. BnPRP1 can be as an anti-infection compound and as a novel class of potential cell-penetrating peptides that could be used to internalize membrane-impermeant drug into both bacterial and fungal cells. The study of this novel plant-derived PR-AMP provides a new chance to improve the resistance against pathogens by the genetic engineering technology. Its biological activity is a basis for development in the pharmaceutical and agricultural fields.

## References

[1] Epand RM, Vogel HJ. Diversity of antimicrobial peptides and their mechanisms of action. Biochim Biophys Acta. 1999; 1462: 11–28. 10590300 10.1016/s0005-2736(99)00198-4

[2] Hwang PM, Vogel HJ. Structure-function relationships of antimicrobial peptides. Biochem Cell Biol. 1998; 76: 235–246. 9923692 10.1139/bcb-76-2-3-235

[3] Ludtke S, He K, Huang H. Membrane thinning caused by magainin 2. Biochemistry. 1995; 34: 16764–16769. 8527451 10.1021/bi00051a026

[4] Chamorro C, Boerman MA, Arnusch CJ, Breukink E, Pieters RJ. Enhancing membrane disruption by targeting and multivalent presentation of antimicrobial peptides. Biochim Biophys Acta. 2012; 1818: 2171–2174. 10.1016/j.bbamem.2012.04.004 22525599

[5] Skerlavaj B, Gennaro R, Bagella L, Merluzzi L, Risso A, Zanetti M. Biological characterization of two novel cathelicidin-derived peptides and identification of structural requirements for their antimicrobial and cell lytic activities. J Biol Chem. 1996; 271: 28375–28381. 8910461 10.1074/jbc.271.45.28375

[6] Otvos L Jr. Antibacterial peptides isolated from insects. J Pept Sci. 2000; 6: 497–511. 11071264 10.1002/1099-1387(200010)6:10<497::AID-PSC277>3.0.CO;2-W

[7] Gennaro R, Zanetti M, Benincasa M, Podda E, Miani M. Pro-rich antimicrobial peptides from animals: structure, biological functions and mechanism of action. Curr Pharm Des. 2002; 8: 763–778. 11945170 10.2174/1381612023395394

[8] Scocchi M, Tossi A, Gennaro R. Proline-rich antimicrobial peptides: converging to a non-lytic mechanism of action. Cell Mol Life Sci. 2011; 68: 2317–2330. 10.1007/s00018-011-0721-7 21594684 PMC11114787

[9] Casteels P, Ampe C, Jacobs F, Vaeck M, Tempst P. Apidaecins: antibacterial peptides from honeybees. EMBO J. 1989; 8: 2387–2391. 2676519 10.1002/j.1460-2075.1989.tb08368.x PMC401180

[10] Li JX, Xu XQ, Yu HN, Yang HL, Huang ZX, Lai R. Direct antimicrobial activities of PR-bombesin. Life Sci. 2006; 78: 1953–1956. 16263139 10.1016/j.lfs.2005.08.034

[11] Destoumieux D, Bulet P, Strub JM, Van Dorsselaer A, Bachère E. Recombinant expression and range of activity of penaeidins, antimicrobial peptides from penaeid shrimp. Eur J Biochem. 1999; 266: 335–346. 10561573 10.1046/j.1432-1327.1999.00855.x

[12] Gueguen Y, Bernard R, Julie F, Paulina S, Delphine DG, Franck V, et al Oyster hemocytes express a proline-rich peptide displaying synergistic antimicrobial activity with a defensin. Mol Immunol. 2009; 46: 516–522. 10.1016/j.molimm.2008.07.021 18962895

[13] Padovan L, Scocchi M, Tossi A. Structural aspects of plant antimicrobial peptides. Curr Protein Pept Sci. 2010; 11: 210–219. 20088769 10.2174/138920310791112093

[14] Nawrot R, Barylski J, Nowicki G, Broniarczyk J, Buchwald W, Goździcka-Józefiak A. Plant antimicrobial peptides. Folia Microbiol (Praha). 2014; 59: 181–196. 24092498 10.1007/s12223-013-0280-4 PMC3971460

[15] Daly NL, Rosengren KJ, Craik DJ. Discovery, structure and biological activities of cyclotides. Adv Drug Deliv Rev. 2009; 61: 918–930. 10.1016/j.addr.2009.05.003 19470399

[16] Wu G, Li X, Fan X, Wu H, Wang S, Shen Z, et al The activity of antimicrobial peptide S-thanatin is independent on multidrug-resistant spectrum of bacteria. Peptides. 2011; 32: 1139–1145. 10.1016/j.peptides.2011.03.019 21453736

[17] Cabras T, Longhi R, Secundo F, Nocca G, Conti S, Polonelli L, et al Structural and functional characterization of the porcine proline-rich antifungal peptide SP-B isolated from salivary gland granules. J Pept Sci. 2008; 14: 251–260. 17883246 10.1002/psc.914

[18] Ke T, Liang S, Huang J, Mao H, Chen JB, Dong CH, et al A novel PCR-based method for high throughput prokaryotic expression of antimicrobial peptide genes. BMC Biotechnol. 2012; 12: 10 10.1186/1472-6750-12-10 22439858 PMC3350388

[19] Brahmachary M, Krishnan SP, Koh JL, Khan AM, Seah SH, Tan TW, et al ANTIMIC: a database of antimicrobial sequences. Nucleic Acids Res. 2004; 32: D586–589. 14681487 10.1093/nar/gkh032 PMC308766

[20] Wang G, Li X, Wang Z. APD2: the updated antimicrobial peptide database and its application in peptide design. Nucleic Acids Res. 2009; 37: D933–937. 10.1093/nar/gkn823 18957441 PMC2686604

[21] Hammami R, Ben Hamida J, Vergoten G, Fliss I. PhytAMP: a database dedicated to antimicrobial plant peptides. Nucleic Acids Res. 2009; 37: D963–968. 10.1093/nar/gkn655 18836196 PMC2686510

[22] Thompson JD, Gibson TJ, Higgins DG. Multiple sequence alignment using ClustalW and ClustalX. Curr Protoc Bioinformatics. 2002; Chapter 2: Unit 2 3. 10.1002/0471250953.bi0203s00 18792934

[23] Tamura K, Peterson D, Peterson N, Stecher G, Nei M, Kumar S. MEGA5: molecular evolutionary genetics analysis using maximum likelihood, evolutionary distance, and maximum parsimony methods. Mol Biol Evol. 2011; 28: 2731–2739. 10.1093/molbev/msr121 21546353 PMC3203626

[24] Schagger H, von Jagow G. Tricine-sodium dodecyl sulfate-polyacrylamide gel electrophoresis for the separation of proteins in the range from 1 to 100 kDa. Anal Biochem. 1987; 166: 368–379. 2449095 10.1016/0003-2697(87)90587-2

[25] Lehrer RI, Rosenman M, Harwig SS, Jackson R, Eisenhauer P. Ultrasensitive assays for endogenous antimicrobial polypeptides. J Immunol Methods. 1991; 137: 167–173. 1901580 10.1016/0022-1759(91)90021-7

[26] Liu Y, Xu Q, Chen Z. Purification and characterization of antifungal peptide LP-1. Wei Sheng Wu Xue Bao. 1999; 39: 441–447. 12555526

[27] Park CB, Yi KS, Matsuzaki K, Kim MS, Kim SC. Structure-activity analysis of buforin II, a histone H2A-derived antimicrobial peptide: the proline hinge is responsible for the cell-penetrating ability of buforin II. Proc Natl Acad Sci U S A. 2000; 97: 8245–8250. 10890923 10.1073/pnas.150518097 PMC26932

[28] Chen YH, Yang JT, Martinez HM. Determination of the secondary structures of proteins by circular dichroism and optical rotatory dispersion. Biochemistry. 1972; 11: 4120–4131. 4343790 10.1021/bi00772a015

[29] Chakchouk-Mtibaa A, Elleuch L, Smaoui S. Najah S, Sellem L, Abdelkafi S, et al An antilisterial bacteriocin BacFL31 produced by *Enterococcus faecium* FL31 with a novel structure containing hydroxyproline residues. Anaerobe. 2014; 27: 1–6. 10.1016/j.anaerobe.2014.02.002 24583094

[30] Casteels P, Ampe C, Riviere L, Van Damme J, Elicone C, A Fleming M . Isolation and characterization of abaecin, a major antibacterial response peptide in the honeybee (*Apis mellifera*) Eur J Biochem. 1990; 26: 381–386. 10.1111/j.1432-1033.1990.tb15315.x 2298215

[31] Rees JA, Moniatte M, Bulet P. Novel antibacterial peptides isolated from a European bumblebee, *Bombus pascuorum* (*Hymenoptera*, *Apoidea*). Insect Biochem Mol Biol. 1997; 27: 413–422. 9219367 10.1016/s0965-1748(97)00013-1

[32] Bulet P, Hetru C, Dimarcq JL, Hoffmann D. Antimicrobial peptides in insects; structure and function. Dev Comp Immunol. 1999; 23: 329–344. 10426426 10.1016/s0145-305x(99)00015-4

[33] Otvos L Jr. The short proline-rich antibacterial peptide family. Cell Mol Life Sci. 2002; 59: 1138–1150. 12222961 10.1007/s00018-002-8493-8 PMC11337521

[34] Boman HG, Agerberth B, Boman A. Mechanisms of action on *Escherichia coli* of cecropin P1 and PR-39, two antibacterial peptides from pig intestine. Infect Immun. 1993; 61: 2978–2984. 8514403 10.1128/iai.61.7.2978-2984.1993 PMC280948

[35] Wessely-Szponder J, Majer-Dziedzic B, Smolira A. Analysis of antimicrobial peptides from porcine neutrophils. J Microbiol Methods. 2010; 83: 8–12. 10.1016/j.mimet.2010.07.010 20643166

[36] Bodkin MJ, Goodfellow JM. Hydrophobic solvation in aqueous trifluoroethanol solution. Biopolymers. 1996; 39: 43–50. 8924626 10.1002/(SICI)1097-0282(199607)39:1%3C43::AID-BIP5%3E3.0.CO;2-V

